# Bis[μ_3_-*N*′-oxidopyridine-2-carbox­imidamidato(2−)]bis­[μ_2_-*N*′-oxido­pyridine-2-carboximidamidato(1−)]tetra­pyridine­tetra­nickel(II) dinitrate

**DOI:** 10.1107/S1600536811052524

**Published:** 2011-12-10

**Authors:** Xiao-Hui Deng, Jing-Wen Ran

**Affiliations:** a Institute of Cash Crops, Hubei Academy of Agricultural Science, Wuhan 430064, People’s Republic of China; b Key Laboratory of Industrial Ecology and Environmental Engineering (MOE) and State Key Laboratory of Fine Chemicals, School of Environmental Science and Technology, Dalian University of Technology, Dalian 116024, People’s Republic of China

## Abstract

The title compound, [Ni_4_(C_6_H_5_N_3_O)_2_(C_6_H_6_N_3_O)_2_(C_5_H_5_N)_4_](NO_3_)_2_, is a tetra­nuclear nickel complex containing a single-decker cation, located on an inversion center. The two unique Ni^II^ cations are *N*,*N*′,*N*′′,*O*-chelated by carbox­imid­amid­ate(2−) and carboximidamidate(1−) anions, forming a distorted four-coordinate planar structure, while the other two Ni^II^ atoms are *N*,*N*′,*O*,*O*′-chelated by the same bridging ligands and two pyridine mol­ecules, affording six-coordinated metals in an octa­hedral geometry. The cation is isostructural with the complex crystallized with perchlorate counter-ions in place of nitrate.

## Related literature

For similar metal complexes, see: Kou *et al.* (2010 ▶); Papatriantafyllopoulou *et al.* (2008 ▶); Inglis *et al.* (2010 ▶); Deng & Ran (2011 ▶). For the synthesis of the ligand pyridine-2-amidoxine, see: Bernasek (1957 ▶).
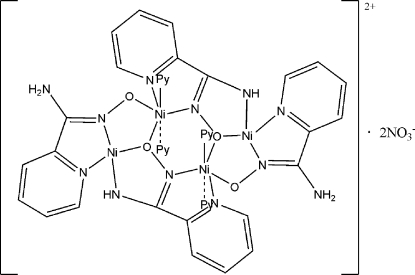


## Experimental

### 

#### Crystal data

[Ni_4_(C_6_H_5_N_3_O)_2_(C_6_H_6_N_3_O)_2_(C_5_H_5_N)_4_](NO_3_)_2_
*M* *_r_* = 1217.80
Triclinic, 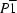

*a* = 10.4356 (6) Å
*b* = 10.7190 (8) Å
*c* = 11.2908 (9) Å
α = 92.041 (6)°
β = 98.240 (5)°
γ = 100.870 (5)°
*V* = 1224.96 (15) Å^3^
*Z* = 1
Mo *K*α radiation
μ = 1.59 mm^−1^
*T* = 293 K
0.40 × 0.38 × 0.35 mm

#### Data collection

Bruker SMART APEXII CCD area-detector diffractometer
Absorption correction: multi-scan (*SADABS*; Bruker, 2007 ▶) *T*_min_ = 0.569, *T*_max_ = 0.606
9481 measured reflections
4286 independent reflections
3180 reflections with *I* > 2σ(*I*)
*R*_int_ = 0.039

#### Refinement

*R*[*F* ^2^ > 2σ(*F* ^2^)] = 0.041
*wR*(*F* ^2^) = 0.100
*S* = 1.03
4286 reflections
349 parameters
8 restraints
H atoms treated by a mixture of independent and constrained refinement
Δρ_max_ = 0.66 e Å^−3^
Δρ_min_ = −0.52 e Å^−3^

### 

Data collection: *APEX2* (Bruker, 2007 ▶); cell refinement: *SAINT* (Bruker, 2007 ▶); data reduction: *SAINT*; program(s) used to solve structure: *SHELXS97* (Sheldrick, 2008 ▶); program(s) used to refine structure: *SHELXL97* (Sheldrick, 2008 ▶); molecular graphics: *SHELXTL* (Sheldrick, 2008 ▶); software used to prepare material for publication: *SHELXTL*.

## Supplementary Material

Crystal structure: contains datablock(s) I, global. DOI: 10.1107/S1600536811052524/bh2397sup1.cif

Structure factors: contains datablock(s) I. DOI: 10.1107/S1600536811052524/bh2397Isup2.hkl

Additional supplementary materials:  crystallographic information; 3D view; checkCIF report

## Figures and Tables

**Fig. 1.**
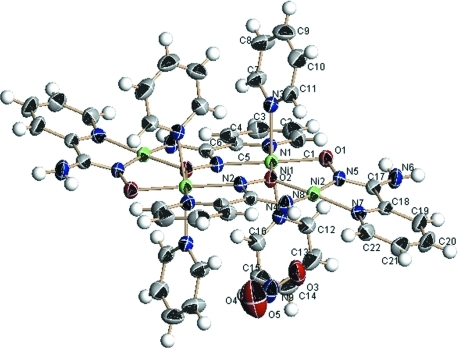
The molecular structure of the title complex, showing displacement ellipsoids at the 50% probability level.

## supplementary crystallographic information

### Comment

Transition metal compounds have been of great interest for many years. They are very important in the development of coordination chemistry. As an extension of work on the structural characterization of Ni compounds, we report here the crystal structure of a new tetranuclear nickel(II) compound.

The title compound is a tetranuclear nickel(II) complex (Fig. 1). The Ni(II) ions form a central deck, but show different coordinate ways. Two are coordinated by N*_py_*, N*_ox_*, N*_am_* and O*_ox_* (where the abbreviations *py*, *ox* and *am* are for the 2-pyridyl, oximate and deprotonated amino donor atoms, respectively), and form square planar geometry. The other two metals are coordinated by two solvent pyridine molecules and two chelating ligands, to form octahedral structures. The different coordinated-nickel(II) ions are bridged by the O*_ox_* groups. The Ni1—(N,O) bond lengths are longer than the Ni2—(N,O) bond distances. The angles are also different, as a consequence of the different coordination geometry. The cation is indeed isostructural with that found in the perchlorate complex previously reported (Kou *et al.*, 2010). Related complexes have also been characterized (Papatriantafyllopoulou *et al.*, 2008; Inglis *et al.*, 2010; Deng & Ran, 2011).

### Experimental

The synthesis of pyridine-2-amidoxine was carried out according to literature (Bernasek, 1957). The title compound was synthesized by adding a solution of Ni(NO_3_)_2_.6H_2_O (290.8 mg, 1 mmol) in H_2_O (20 ml) to a solution of the ligand (137 mg, 1 mmol) and NaOH (80 mg, 2 mmol) in ethanol/water (3:1, 20 ml). The mixture was stirred at room temperature. The resulting precipitate was collected and dissolved in a mixture of ethanol and pyridine (3:1 *v*/*v*) at 50°C. The solution was allowed to stand in air for one day, and brown crystals were formed at the bottom of the vessel on slow evaporation of the solvent at room temperature. Yield: 56%. Anal. Calcd for C_44_H_42_N_18_Ni_4_O_10_: C 43.40, H 3.48, N 20.70. Found: C 43.36, H 3.53, N 20.94. IR (KBr, cm-^1^): ν = 3412 (w), 3268 (w), 1605 (s), 1552 (s), 1390 (very strong), 1347 (very strong), 1107 (m), 1450 (very strong), 705 (m).

### Refinement

H atoms were included in calculated positions with C—H = 0.93, N—H = 0.86 Å. For the terminal amine group N6, H atoms were refined freely, with restrained N—H bond lengths. Other H atoms were refined using a riding-model. Isotropic displacement parameters for H atoms were calculated as *U*_iso_(H) = 1.2 *U*_eq_(C,N). Anisotropic displacement parameters for nitrate O atoms were restrained.

### Crystal data

**Table:**

[Ni_4_(C_6_H_5_N_3_O)_2_(C_6_H_6_N_3_O)_2_(C_5_H_5_N)_4_](NO_3_)_2_	*Z* = 1
*M**_r_* = 1217.80	*F*(000) = 624
Triclinic, *P*1	*D*_x_ = 1.651 Mg m^−^^3^
Hall symbol: -P 1	Mo *K*α radiation, λ = 0.71073 Å
*a* = 10.4356 (6) Å	Cell parameters from 1738 reflections
*b* = 10.7190 (8) Å	θ = 3.1–21.8°
*c* = 11.2908 (9) Å	µ = 1.59 mm^−^^1^
α = 92.041 (6)°	*T* = 293 K
β = 98.240 (5)°	Block, brown
γ = 100.870 (5)°	0.40 × 0.38 × 0.35 mm
*V* = 1224.96 (15) Å^3^	

### Data collection

**Table:**

Bruker SMART APEXII CCD area-detector diffractometer	4286 independent reflections
Radiation source: fine-focus sealed tube	3180 reflections with *I* > 2σ(*I*)
graphite	*R*_int_ = 0.039
φ and ω scans	θ_max_ = 25.0°, θ_min_ = 2.5°
Absorption correction: multi-scan (*SADABS*; Bruker, 2007)	*h* = −12→12
*T*_min_ = 0.569, *T*_max_ = 0.606	*k* = −12→11
9481 measured reflections	*l* = −9→13

### Refinement

**Table:**

Refinement on *F*^2^	Primary atom site location: structure-invariant direct methods
Least-squares matrix: full	Secondary atom site location: difference Fourier map
*R*[*F*^2^ > 2σ(*F*^2^)] = 0.041	Hydrogen site location: inferred from neighbouring sites
*wR*(*F*^2^) = 0.100	H atoms treated by a mixture of independent and constrained refinement
*S* = 1.03	*w* = 1/[σ^2^(*F*_o_^2^) + (0.042*P*)^2^ + 0.4034*P*] where *P* = (*F*_o_^2^ + 2*F*_c_^2^)/3
4286 reflections	(Δ/σ)_max_ = 0.001
349 parameters	Δρ_max_ = 0.66 e Å^−^^3^
8 restraints	Δρ_min_ = −0.52 e Å^−^^3^
0 constraints	

### Fractional atomic coordinates and isotropic or equivalent isotropic displacement parameters (Å^2^)

**Table:**

	*x*	*y*	*z*	*U*_iso_*/*U*_eq_	
Ni1	0.45373 (3)	0.49188 (3)	0.66926 (3)	0.03132 (8)	
Ni2	0.37394 (3)	0.76610 (3)	0.59280 (3)	0.03324 (9)	
N1	0.45234 (18)	0.33873 (19)	0.77743 (18)	0.0346 (5)	
N2	0.50204 (18)	0.35800 (18)	0.55758 (17)	0.0323 (5)	
N3	0.65769 (18)	0.5669 (2)	0.75316 (18)	0.0364 (6)	
N4	0.24626 (18)	0.4257 (2)	0.61209 (18)	0.0371 (6)	
N5	0.35016 (18)	0.70397 (19)	0.74148 (18)	0.0340 (5)	
N6	0.2507 (2)	0.7224 (2)	0.9124 (2)	0.0510 (7)	
H6B	0.2906 (19)	0.6662 (16)	0.941 (2)	0.061*	
H6A	0.235 (2)	0.7699 (19)	0.9683 (17)	0.061*	
N7	0.27188 (19)	0.88369 (19)	0.63555 (19)	0.0384 (6)	
N8	0.4142 (2)	0.82419 (19)	0.44897 (19)	0.0419 (6)	
H8	0.3973	0.8940	0.4213	0.050*	
N9	0.2065 (2)	0.9041 (2)	0.1685 (2)	0.0626 (7)	
O1	0.40271 (16)	0.60905 (16)	0.79192 (15)	0.0387 (5)	
O2	0.45424 (15)	0.63556 (15)	0.55536 (14)	0.0346 (4)	
O3	0.2335 (2)	0.9647 (2)	0.2650 (2)	0.0902 (8)	
O4	0.1740 (3)	0.7894 (3)	0.1607 (3)	0.1234 (12)	
O5	0.2296 (4)	0.9553 (3)	0.0776 (2)	0.1285 (11)	
C1	0.4176 (3)	0.3303 (3)	0.8866 (2)	0.0477 (8)	
H1	0.3974	0.4017	0.9234	0.057*	
C2	0.4105 (3)	0.2203 (3)	0.9472 (3)	0.0562 (9)	
H2	0.3853	0.2171	1.0229	0.067*	
C3	0.4416 (3)	0.1151 (3)	0.8929 (3)	0.0532 (9)	
H3	0.4352	0.0390	0.9307	0.064*	
C4	0.4823 (2)	0.1235 (3)	0.7816 (2)	0.0410 (7)	
H4	0.5067	0.0544	0.7447	0.049*	
C5	0.4859 (2)	0.2373 (2)	0.7266 (2)	0.0332 (7)	
C6	0.5271 (2)	0.2555 (2)	0.6071 (2)	0.0321 (6)	
C7	0.7375 (2)	0.4927 (3)	0.8027 (3)	0.0480 (8)	
H7	0.7126	0.4051	0.7865	0.058*	
C8	0.8551 (3)	0.5403 (3)	0.8767 (3)	0.0612 (10)	
H8A	0.9077	0.4856	0.9101	0.073*	
C9	0.8934 (3)	0.6691 (3)	0.9002 (3)	0.0651 (11)	
H9	0.9709	0.7033	0.9517	0.078*	
C10	0.8154 (3)	0.7466 (3)	0.8465 (3)	0.0587 (10)	
H10	0.8405	0.8347	0.8585	0.070*	
C11	0.6988 (2)	0.6920 (3)	0.7743 (3)	0.0445 (8)	
H11	0.6461	0.7455	0.7385	0.053*	
C12	0.1604 (2)	0.4086 (3)	0.6893 (3)	0.0497 (8)	
H12	0.1919	0.4291	0.7703	0.060*	
C13	0.0283 (3)	0.3623 (3)	0.6558 (3)	0.0654 (10)	
H13	−0.0283	0.3513	0.7128	0.079*	
C14	−0.0189 (3)	0.3328 (3)	0.5370 (4)	0.0708 (11)	
H14	−0.1084	0.3014	0.5114	0.085*	
C15	0.0670 (3)	0.3500 (3)	0.4571 (3)	0.0664 (10)	
H15	0.0371	0.3309	0.3757	0.080*	
C16	0.1994 (3)	0.3960 (3)	0.4975 (3)	0.0503 (8)	
H16	0.2577	0.4065	0.4419	0.060*	
C17	0.2787 (2)	0.7604 (2)	0.8038 (2)	0.0374 (7)	
C18	0.2286 (2)	0.8621 (2)	0.7436 (2)	0.0368 (7)	
C19	0.1439 (2)	0.9336 (3)	0.7852 (3)	0.0529 (9)	
H19	0.1137	0.9175	0.8577	0.063*	
C20	0.1053 (3)	1.0286 (3)	0.7175 (3)	0.0617 (10)	
H20	0.0498	1.0780	0.7446	0.074*	
C21	0.1495 (3)	1.0495 (3)	0.6102 (3)	0.0585 (9)	
H21	0.1239	1.1132	0.5639	0.070*	
C22	0.2319 (3)	0.9763 (3)	0.5708 (3)	0.0488 (8)	
H22	0.2608	0.9911	0.4975	0.059*	

### Atomic displacement parameters (Å^2^)

**Table:**

	*U*^11^	*U*^22^	*U*^33^	*U*^12^	*U*^13^	*U*^23^
Ni1	0.03287 (15)	0.03169 (17)	0.03126 (17)	0.01043 (13)	0.00592 (13)	0.00101 (13)
Ni2	0.03572 (15)	0.03221 (17)	0.03383 (17)	0.01353 (13)	0.00404 (14)	−0.00190 (14)
N1	0.0353 (10)	0.0386 (12)	0.0312 (11)	0.0102 (9)	0.0055 (9)	0.0022 (9)
N2	0.0389 (10)	0.0305 (11)	0.0292 (11)	0.0082 (9)	0.0091 (9)	0.0017 (9)
N3	0.0362 (10)	0.0368 (11)	0.0378 (12)	0.0088 (9)	0.0088 (9)	0.0010 (10)
N4	0.0353 (10)	0.0417 (12)	0.0359 (11)	0.0088 (9)	0.0094 (9)	0.0008 (10)
N5	0.0349 (10)	0.0352 (11)	0.0342 (11)	0.0131 (9)	0.0061 (9)	−0.0035 (9)
N6	0.0580 (12)	0.0633 (15)	0.0413 (13)	0.0282 (11)	0.0185 (11)	0.0009 (12)
N7	0.0382 (10)	0.0358 (11)	0.0401 (12)	0.0112 (9)	−0.0011 (10)	−0.0057 (10)
N8	0.0565 (12)	0.0310 (11)	0.0451 (13)	0.0210 (10)	0.0133 (10)	0.0057 (10)
N9	0.0837 (15)	0.0704 (16)	0.0504 (15)	0.0439 (13)	0.0246 (13)	0.0175 (13)
O1	0.0458 (9)	0.0395 (10)	0.0345 (9)	0.0176 (8)	0.0066 (8)	0.0013 (8)
O2	0.0448 (8)	0.0347 (9)	0.0299 (9)	0.0180 (7)	0.0107 (7)	0.0037 (7)
O3	0.1267 (16)	0.1144 (18)	0.0461 (13)	0.0744 (14)	0.0053 (13)	−0.0103 (13)
O4	0.151 (2)	0.079 (2)	0.138 (3)	−0.0138 (19)	0.064 (2)	0.0039 (19)
O5	0.243 (3)	0.0930 (18)	0.0743 (17)	0.0774 (19)	0.044 (2)	0.0252 (15)
C1	0.0498 (14)	0.0572 (18)	0.0381 (15)	0.0145 (13)	0.0081 (13)	0.0043 (14)
C2	0.0515 (15)	0.085 (2)	0.0355 (15)	0.0151 (16)	0.0099 (13)	0.0193 (15)
C3	0.0510 (15)	0.0552 (18)	0.0529 (17)	0.0102 (14)	0.0022 (14)	0.0224 (14)
C4	0.0394 (13)	0.0388 (15)	0.0435 (15)	0.0086 (12)	−0.0005 (12)	0.0071 (12)
C5	0.0303 (11)	0.0332 (13)	0.0347 (14)	0.0042 (10)	0.0028 (11)	0.0020 (11)
C6	0.0332 (11)	0.0274 (13)	0.0346 (13)	0.0061 (10)	0.0017 (11)	−0.0005 (11)
C7	0.0384 (13)	0.0400 (15)	0.0642 (19)	0.0097 (12)	0.0004 (14)	0.0037 (14)
C8	0.0447 (15)	0.0564 (18)	0.078 (2)	0.0122 (14)	−0.0111 (16)	0.0107 (17)
C9	0.0421 (15)	0.068 (2)	0.074 (2)	0.0004 (16)	−0.0103 (16)	−0.0087 (18)
C10	0.0466 (15)	0.0435 (17)	0.081 (2)	0.0018 (14)	0.0066 (16)	−0.0128 (16)
C11	0.0408 (13)	0.0436 (15)	0.0519 (17)	0.0136 (12)	0.0096 (13)	0.0020 (13)
C12	0.0398 (13)	0.0613 (18)	0.0474 (17)	0.0088 (13)	0.0082 (13)	−0.0031 (15)
C13	0.0443 (15)	0.060 (2)	0.096 (3)	0.0107 (14)	0.0270 (16)	−0.0007 (19)
C14	0.0370 (14)	0.0539 (19)	0.115 (3)	0.0073 (14)	−0.0030 (17)	−0.021 (2)
C15	0.0554 (17)	0.069 (2)	0.066 (2)	0.0117 (16)	−0.0144 (16)	−0.0184 (18)
C16	0.0502 (15)	0.0569 (18)	0.0431 (16)	0.0117 (14)	0.0049 (13)	−0.0024 (14)
C17	0.0351 (12)	0.0359 (14)	0.0399 (15)	0.0069 (11)	0.0032 (11)	−0.0039 (12)
C18	0.0296 (11)	0.0358 (14)	0.0429 (15)	0.0048 (11)	0.0025 (11)	−0.0080 (12)
C19	0.0429 (13)	0.0549 (17)	0.066 (2)	0.0190 (13)	0.0163 (14)	−0.0062 (15)
C20	0.0542 (15)	0.0575 (18)	0.083 (2)	0.0327 (14)	0.0162 (16)	−0.0032 (17)
C21	0.0596 (15)	0.0522 (17)	0.070 (2)	0.0339 (13)	0.0009 (16)	0.0024 (16)
C22	0.0548 (15)	0.0467 (16)	0.0481 (17)	0.0232 (13)	0.0015 (14)	0.0013 (14)

### Geometric parameters (Å, °)

**Table:**

Ni1—O1	2.0295 (17)	C2—H2	0.9300
Ni1—O2	2.0410 (17)	C3—C4	1.383 (4)
Ni1—N2	2.057 (2)	C3—H3	0.9300
Ni1—N1	2.080 (2)	C4—C5	1.386 (4)
Ni1—N4	2.1435 (19)	C4—H4	0.9300
Ni1—N3	2.1900 (18)	C5—C6	1.481 (3)
Ni2—O2	1.8278 (17)	C6—N8^i^	1.333 (3)
Ni2—N8	1.836 (2)	C7—C8	1.381 (4)
Ni2—N5	1.860 (2)	C7—H7	0.9300
Ni2—N7	1.888 (2)	C8—C9	1.368 (4)
N1—C1	1.335 (3)	C8—H8A	0.9300
N1—C5	1.339 (3)	C9—C10	1.367 (4)
N2—C6	1.303 (3)	C9—H9	0.9300
N2—O2^i^	1.414 (3)	C10—C11	1.378 (4)
N3—C11	1.331 (3)	C10—H10	0.9300
N3—C7	1.336 (3)	C11—H11	0.9300
N4—C16	1.321 (3)	C12—C13	1.367 (4)
N4—C12	1.330 (3)	C12—H12	0.9300
N5—C17	1.306 (3)	C13—C14	1.366 (5)
N5—O1	1.350 (3)	C13—H13	0.9300
N6—C17	1.360 (3)	C14—C15	1.354 (5)
N6—H6B	0.843 (15)	C14—H14	0.9300
N6—H6A	0.851 (16)	C15—C16	1.380 (4)
N7—C22	1.347 (3)	C15—H15	0.9300
N7—C18	1.373 (3)	C16—H16	0.9300
N8—C6^i^	1.333 (3)	C17—C18	1.446 (4)
N8—H8	0.8600	C18—C19	1.391 (4)
N9—O4	1.209 (4)	C19—C20	1.381 (4)
N9—O5	1.214 (4)	C19—H19	0.9300
N9—O3	1.217 (3)	C20—C21	1.368 (5)
O2—N2^i^	1.414 (3)	C20—H20	0.9300
C1—C2	1.380 (4)	C21—C22	1.376 (4)
C1—H1	0.9300	C21—H21	0.9300
C2—C3	1.378 (4)	C22—H22	0.9300
			
O1—Ni1—O2	87.43 (7)	C2—C3—H3	120.2
O1—Ni1—N2	173.84 (8)	C4—C3—H3	120.2
O2—Ni1—N2	98.68 (7)	C3—C4—C5	118.3 (3)
O1—Ni1—N1	95.46 (8)	C3—C4—H4	120.8
O2—Ni1—N1	176.94 (7)	C5—C4—H4	120.8
N2—Ni1—N1	78.42 (8)	N1—C5—C4	122.4 (2)
O1—Ni1—N4	86.65 (7)	N1—C5—C6	115.2 (2)
O2—Ni1—N4	90.52 (7)	C4—C5—C6	122.4 (2)
N2—Ni1—N4	92.45 (8)	N2—C6—N8^i^	120.5 (2)
N1—Ni1—N4	88.65 (8)	N2—C6—C5	115.1 (2)
O1—Ni1—N3	85.71 (7)	N8^i^—C6—C5	124.5 (2)
O2—Ni1—N3	92.71 (7)	N3—C7—C8	123.0 (3)
N2—Ni1—N3	94.77 (8)	N3—C7—H7	118.5
N1—Ni1—N3	88.52 (7)	C8—C7—H7	118.5
N4—Ni1—N3	171.56 (8)	C9—C8—C7	119.1 (3)
O2—Ni2—N8	84.38 (8)	C9—C8—H8A	120.5
O2—Ni2—N5	91.50 (8)	C7—C8—H8A	120.5
N8—Ni2—N5	174.27 (9)	C10—C9—C8	118.7 (3)
O2—Ni2—N7	172.23 (8)	C10—C9—H9	120.7
N8—Ni2—N7	100.57 (9)	C8—C9—H9	120.7
N5—Ni2—N7	83.96 (9)	C9—C10—C11	118.9 (3)
C1—N1—C5	118.4 (2)	C9—C10—H10	120.6
C1—N1—Ni1	127.43 (19)	C11—C10—H10	120.6
C5—N1—Ni1	114.07 (16)	N3—C11—C10	123.4 (3)
C6—N2—O2^i^	109.62 (19)	N3—C11—H11	118.3
C6—N2—Ni1	115.76 (16)	C10—C11—H11	118.3
O2^i^—N2—Ni1	132.58 (14)	N4—C12—C13	123.4 (3)
C11—N3—C7	116.9 (2)	N4—C12—H12	118.3
C11—N3—Ni1	119.41 (17)	C13—C12—H12	118.3
C7—N3—Ni1	122.75 (16)	C14—C13—C12	118.7 (3)
C16—N4—C12	117.3 (2)	C14—C13—H13	120.7
C16—N4—Ni1	120.50 (18)	C12—C13—H13	120.7
C12—N4—Ni1	122.16 (16)	C15—C14—C13	118.7 (3)
C17—N5—O1	117.4 (2)	C15—C14—H14	120.7
C17—N5—Ni2	116.46 (18)	C13—C14—H14	120.7
O1—N5—Ni2	126.15 (15)	C14—C15—C16	119.5 (3)
C17—N6—H6B	115.2 (18)	C14—C15—H15	120.2
C17—N6—H6A	125.6 (16)	C16—C15—H15	120.2
H6B—N6—H6A	111 (2)	N4—C16—C15	122.4 (3)
C22—N7—C18	118.8 (2)	N4—C16—H16	118.8
C22—N7—Ni2	128.23 (19)	C15—C16—H16	118.8
C18—N7—Ni2	112.85 (17)	N5—C17—N6	122.5 (2)
C6^i^—N8—Ni2	111.45 (17)	N5—C17—C18	113.5 (2)
C6^i^—N8—H8	124.3	N6—C17—C18	123.9 (2)
Ni2—N8—H8	124.3	N7—C18—C19	120.8 (3)
O4—N9—O5	116.9 (3)	N7—C18—C17	113.1 (2)
O4—N9—O3	121.9 (3)	C19—C18—C17	126.1 (3)
O5—N9—O3	120.3 (3)	C20—C19—C18	119.3 (3)
N5—O1—Ni1	112.62 (13)	C20—C19—H19	120.4
N2^i^—O2—Ni2	114.03 (13)	C18—C19—H19	120.4
N2^i^—O2—Ni1	127.93 (13)	C21—C20—C19	119.4 (3)
Ni2—O2—Ni1	117.72 (8)	C21—C20—H20	120.3
N1—C1—C2	122.7 (3)	C19—C20—H20	120.3
N1—C1—H1	118.6	C20—C21—C22	120.0 (3)
C2—C1—H1	118.6	C20—C21—H21	120.0
C3—C2—C1	118.5 (3)	C22—C21—H21	120.0
C3—C2—H2	120.8	N7—C22—C21	121.8 (3)
C1—C2—H2	120.8	N7—C22—H22	119.1
C2—C3—C4	119.5 (3)	C21—C22—H22	119.1

Symmetry codes: (i) −*x*+1, −*y*+1, −*z*+1.
